# Effect of Ginseng Supplementation on Exercise Endurance as a Support for Cardiovascular Disease Management: A Systematic Review and Meta-Analysis

**DOI:** 10.3390/antiox14010032

**Published:** 2024-12-29

**Authors:** Angelika Szymańska, Anna Nowak, Anna Lipert, Ewa Kochan

**Affiliations:** 1 Department of Pharmacological Biotechnology, Medical University of Lodz, 90-152 Lodz, Poland; 2 Department of Preventive Medicine, Medical University of Lodz, 92-213 Lodz, Poland; anna.nowak10@stud.umed.lodz.pl (A.N.); anna.lipert@umed.lodz.pl (A.L.)

**Keywords:** ginseng, supplement, physical activity, cardiovascular system

## Abstract

Ginseng has multi-directional pharmacological properties. Some data suggest that ginseng can enhance physical endurance, which, in turn, leads to protection of the cardiovascular system. However, not all experiments are conclusive. For this reason, the main aim of this research was to perform a meta-analysis and review of studies published between the years 2013 and 2023 concerning the ginseng effect on physical performance in animal and human models. Medline, Pubmed, and ClinicalKey electronic databases were used to analyze data. The search strategy included the following criteria: ginseng and exercise; ginseng supplementation; and ginseng supplements. The results suggest that ginseng supplementation may have a positive effect on CK levels in animal studies. Similar observations were stated in relation to serum lactate and BUN. Furthermore, a human study showed a significant increase in exercise time to exhaustion and VO2 max after supplementation. The review of the literature and conducted meta-analysis identified that ginseng supplementation may have a positive effect on exercise endurance. Due to the fact that most of the current studies were based on animal models, further research on human models is needed to identify the most effective dosage or form of applied ginseng to be a supportive element in CVD management.

## 1. Introduction

Ginseng is a general name for the plant genus *Panax*, which includes eleven species such as *P. ginseng*, *P. quinquefolius*, *P. notoginseng*, *P. pseudoginseng*, *P. trifolius*, *P. zingiberensis*, *P. stipuleanatus*, *P. japonicus*, *P. japonicus* var. *angustifolius*, *P. japonicus* var. *major*, and *P. japonicus* var. *Bipinnatifidus* [1]. Most of them are used in traditional and natural medicine in various forms, such as whole root, root powder, teas, tinctures, as well as standardized root extracts [2]. Different studies and randomized controlled trials demonstrated that *Panax ginseng* (Asian ginseng) and *Panax quinquefolium* (American ginseng) are the two most commonly investigated species of ginseng for their pharmacological properties [3,4,5,6]. They have also been included in the Chinese and European Pharmacopoeias [7]. Over the centuries, various studies have been conducted to demonstrate the pharmacological properties of ginseng. There is evidence that shows its anti-inflammatory, liver-protective, immune system-boosting effects. In addition, there are also indications of anticancer, antiviral, immunomodulatory, antioxidant properties, and cardiovascular protection [8]. Plants of the *Panax* genus regulate the action of nervous and endocrine systems. In addition, they are characterized as tonic plants whose task is to maintain homeostasis and increase the body’s resistance to all negative factors. These actions can be described as anti-fatigue, anti-aging, and improving the overall vitality of the body [9,10]. The literature data show that diverse classes of biologically active compounds have been isolated from plants of the genus *Panax*, including ginsenosides, polysaccharides, polyacetylenes, and amino acids. Ginsenosides are found to be responsible for most pharmacological activity of ginseng [11]. Currently, about 289 of these metabolites are known [12]. From a chemical point of view, ginsenosides are glycosides containing a non-sugar part in their structure, i.e., aglycone and a sugar chain.

There are three main types of aglycones: (a) tetracyclic type of dammarate; (b) pentacyclic type of oleanolic acid; and (c) tetracyclic type of ocotillol. The sugar part may include, for example, hexoses, 6-deoxyhexoses, pentoses and uronic acids [12]. Due to the content of hydroxyl groups, ginsenosides can be divided into two main categories: the first one is protopanaxadiols (PPD), and the second one is protopanaxatriols (PPT) [13,14]. The group of protopanaxadiols includes ginsenosides such as Rb1, Rc, Rb2, and Rd, while the group of protopanaxatriols includes Rg1 and Re [15]. The quantitative content of ginsenosides depends on many factors, such as the ginseng species, age and part of the plant, and the region of cultivation [16,17,18]. For example, New Zealand’s forest-grown *Panax ginseng* and *Panax quinquefolium* L. accumulated the highest amount of triterpene saponins in fine roots, compared to other tissues, at level 142.49 ± 1.14 and 115.69 ± 3.51 mg/g d.w. respectively, with Rb1 being the most abundant compound. In above-ground parts, such as stem and leaves, ginsenoside Re was quantitatively dominant in both species [17].

Meanwhile, Hou et al. (2021) showed that the ginsenoside quantity could be 0.06 mg/g in *P. trifolius*, 5.8–15.6 mg/g in *P. ginseng*, 55.0–70.4 mg/g in *P. notoginseng*, 68.1–167.1 mg/g in *P. vietnamensis*, and 192.8–296.2 mg/g in *P. japonicus*. In addition, the levels of particular ginsenosides differed in a single species [18].

Many experiments, carried out both in vitro and in vivo conditions, proved the individual effectiveness among ginsenosides. For example, panaxoside Rb1 has antioxidant, anti-inflammatory, immunomodulatory, and neuroprotective effects and also alleviates kidney damage. It has also been proven that it has protective properties for human vascular smooth muscle cells and that it delays the aging and dysfunction of these cells by reducing oxidative stress. Recently, it has also been proven that it can be used in the treatment of neurological diseases, as it protects the neurons and improves their regeneration. Therefore, it has properties that are used to support the treatment of diseases of the nervous, cardiovascular, and hormonal systems [19,20,21]. Panaxoside Rh2 alleviates heart muscle damage caused by excessively high levels of angiotensin-2 [22].

Ginsenoside Rg2 has a cardioprotective effect, and there is even potential to use it in the treatment of ischemic heart disease. It reduces the heart rate in rats and increases the viability of cardiomyocytes. Similarly to Rb1, it also has neuroprotective, antidiabetic, and antiatherosclerotic properties. Moreover, ginsenoside Rg2 lowers cholesterol and triglyceride levels and stimulates lipolysis [13,23,24].

Panaxoside Rc alleviates lipid metabolism disorders, reduces lipogenesis, regulates blood glucose levels, has an anti-inflammatory effect, lowers cholesterol levels, has a hepatoprotective effect, and inhibits the degradation of articular cartilage. It also alleviates the negative effects of ischemic heart damage through its antioxidant effect, which also helps to alleviate oxidative stress occurring in skeletal muscles [25,26]. Rc has a neuroprotective effect in neurodegenerative diseases, e.g., Alzheimer’s disease; it has a soothing effect on the symptoms of multiple sclerosis; it also has an anticonvulsant effect, and, similarly to other ginsenosides, it has cardioprotective and antioxidant effects [24,27,28]. Ginsenosides Rg1 and Rg3 reduce the level of COX-2, inhibit the release of histamine, relax smooth muscles, and dilate blood vessels, thus having a hypotensive effect. Rg1 prevents muscle atrophy by inhibiting protein degradation via the PI3K/Akt/FoxO signaling pathways and promoting protein synthesis via the Akt/mTOR signaling pathway. Additionally, it lowers blood glucose levels and stabilizes heart function. Saponin Rg3 decreases muscle atrophy induced by glucocorticoid and TNF-α by enhancing mitochondrial function [29], while Rd ameliorates aging- and cancer-induced muscle wasting [30].

**Figure 1:**
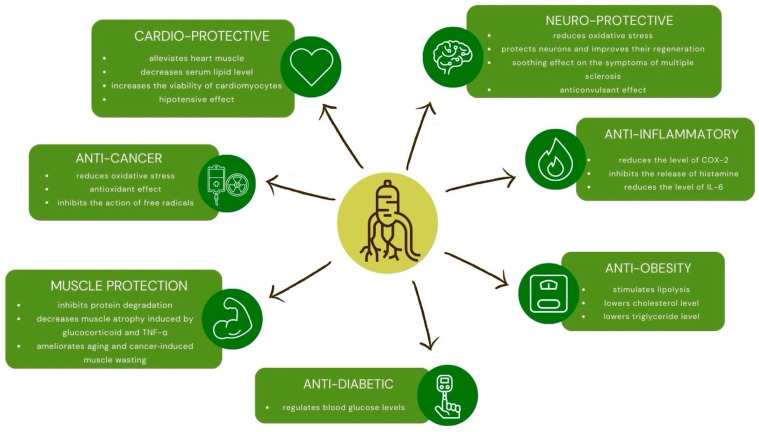
Pharmacological properties of ginsenosides (source: authors’ own work).

All the properties mentioned in Figure 1 perfectly document the pleiotropic and unique effects of ginsenosides, giving the base of potential ability to improve also physical performance, which ultimately may lead to improved functioning of the cardiovascular system, reduced risk of developing hypertension, and even reduced mortality rate.

Muscular endurance refers to a muscle’s ability to work over a period of time and it is closely connected with the term “physical activity” (PA). PA consists of the activity of skeletal muscles, various systems (supplying the muscles with energy substrates and oxygen), and regulatory processes maintaining homeostasis in the body [31]. The cardiovascular system (CVS) consists of the heart, arteries, veins, and capillaries. It is regulated by various mechanisms that help integrate all of its parts. CVS can be influenced, among other factors, by blood volume, medications, and physical activity [32,33]. In 2010, the American Heart Association (AHA), apart from addressing CVD and its risk factors, added different strategies that could affect and promote peoples’ health and created the so-called Life’s Essential 8. It is a new approach to not only measuring but also monitoring and, most importantly, modifying cardiovascular health (CVH). It consists of eight extremely important components for CVH, which also include PA [34]. PA is said to contribute to promoting systemic blood circulation, enhancing mitochondrial function and energy metabolism in skeletal muscle cells, and also maintaining an antiatherosclerotic composition. Engaging in regular moderate physical activity is crucial for reducing both the severity and the likelihood of recurrence of heart disease [35]. PA may help prevent hypertension by affecting the sympathetic nervous system activity, the renin–angiotensin system, sodium handling, and improving endothelial function, rather than relying on weight management. Even a slight increase in physical activity may have a positive impact on health and reduce mortality rates. Additionally, exercise can help lower the risk of diabetes by boosting insulin sensitivity and glucose uptake in skeletal muscle. Activities such as aerobic or endurance exercises are responsible for lowering high blood pressure and altering the lipid profile by reducing triglyceride and LDL levels while increasing HDL levels. Healthy bone mass and overall bone health can be maintained by exercises such as skipping or weight training [36,37].

The muscle cells can be monitored based on the determination of intracellular compounds released into the blood as a result of this damage. Usually, the indicators of muscle damage are concentrations of enzymes such as creatine kinase (CK) or lactate dehydrogenase (LDH) [38].

CK, an enzyme contained in skeletal and cardiac muscle cells, catalyzes energy reactions. It is achieved by moving phosphate from creatine and adenosine diphosphate to generate two products—ATP and creatine. As a result, ATP becomes available for the muscle contractions. CK has three cytoplasmic isoforms: CK-MM (dominantly in muscle fibers in areas with high ATP consumption); CK-MB (mainly in the myocardial muscle); and CK-BB (mainly in the brain) [39]. Creatine kinase in muscle cells catalyzes the reversible phosphorylation of creatine to phosphocreatine or ADP to ATP [40]. Intense long-term exercise, as well as eccentric muscular training, can result in perforations in the sarcolemma and damage to sarcomeres. Increasing membrane permeability due to damaging the muscle structures releases CK into the circulation [39,41]. Levels of CK can vary depending on race and sex due to differences in muscle and total body mass [42]. The time it takes for CK levels in the blood to reach their maximum may differ depending on the type of exercise [43]. A decrease in work output alongside fatigue can be connected with elevated creatine kinase levels [44].

Lactic acid, also known as lactate, is generated from pyruvate through glycolysis. The metabolic fate of lactate depends on factors like the current energy state of the entire body, tissue oxygenation, and the degree and direction of adaptation to various types of physical exercise [45]. During low-impact exercise, the blood lactate concentration increases only slightly or does not change at all [46]. Intense exercise, which restricts the cellular oxygen supply, leads to an increase in lactate production and a pH decrease in active muscle. Before being converted to lactate, pyruvate can follow one of two paths. Firstly, it can be utilized in mitochondria, converted to acetyl-coenzyme A (by pyruvate dehydrogenase—PDH), and used for oxidative phosphorylation in the TCA cycle. This path allows PDH to irreversibly remove lactate. Pyruvate can also be converted to L-lactate (by lactate dehydrogenase—LDH) in anaerobic glycolysis. Lactate can be used in the skeletal muscles for glycogen resynthesis [41,47,48,49]. A mechanism called the lactate shuttle allows different tissues to share lactate as a carbon source for oxidation, fuel energy source, signaling, and other processes [50]. Lactate blood concentration can be used to determine the lactate threshold. The lactate threshold (or anaerobic threshold) is a submaximal load at which the share of anaerobic processes in the metabolism of working muscles increases significantly [31].

Due to the desire to accelerate recovery after exercise, reduce muscle damage, or improve physical performance, natural products are applied as dietary supplements. The effects of a great amount of supplements also depend on the type of exercise. Despite their popularity, many supplements have little to no effect on the recovery process after exercise. For example, small effects of a single supplementation of cannabidiol supplements on CK and myoglobin concentrations were observed 72 h after complex training [51]. Strong evidence was found regarding the ergogenic effect of creatine monohydrate [52] and a possible increase in maximal muscular strength and reduction in muscle damage [53]. As for spirulina supplementation, it has beneficial effects on not only muscle performance but also muscle damage [54]. Despite the fact that there are many substances available on the market that are dedicated to athletes, research shows that their effect is negligible or non-existent. For this reason, further potential supplements that could solve the problem of prolonged recovery times after training, muscle damage, and improving overall physical performance should still be sought. Such a supplement seems to be ginseng plants.

The aim of the present study was to perform a systematic review of the existing literature and subsequently apply a meta-analysis to identify the effect of ginseng supplementation on exercise endurance. We hypothesized that ginseng supplementation enhancing at least some parameters may be proven useful in CVD management.

## 2. Materials and Methods

### 2.1. Database and Searching Strategy

A systematic review was conducted to assess the effect of ginseng supplementation on parameters related to physical performance among animals and humans. The inclusion criteria for the review contained (a) studies conducted on humans, (b) studies conducted on mice or rats, (c) studies conducted using plants of the genus *Panax*, and (d) studies in which all the above criteria and exercise endurance parameters were met.

This review included all the English-language literature from January 2012 to November 2023. Medline, Pubmed, and ClinicalKey are electronic databases that were incorporated into this review. The search strategy was based on the use of three combinations: (a) ginseng and exercise; (b) ginseng supplementation; and (c) ginseng supplements. Studies involving apparently healthy humans, mice, and rats were searched for publication.

### 2.2. Exclusion Criteria

The search results excluded publications on ginseng supplementation among humans and animals with chronic diseases and accompanying symptoms. Thus, the first phase of this review was to exclude papers that involved humans or animals suffering from diseases and symptoms such as diabetes, hyperlipidemia, hypertension, various types of cancer, neurodegenerative diseases, respiratory infections, impotence, menopause, chronic fatigue, obesity, liver damage, stress at work.

The exclusion criteria also included publications in which ginseng was not the only supplemented plant and the studies in which, apart from compounds derived from *Panax* species, subjects also received other substances.

At this stage of work, studies in which parameters directly or indirectly related to physical performance were examined but were different than those included in this review, were also rejected. In the second phase of this research, papers that were duplicated in two or each of the searched databases were also rejected. However, the main reason for exclusion was the occurrence of chronic diseases and other symptoms in both humans and animals.

### 2.3. Search Results

**Figure 2:**
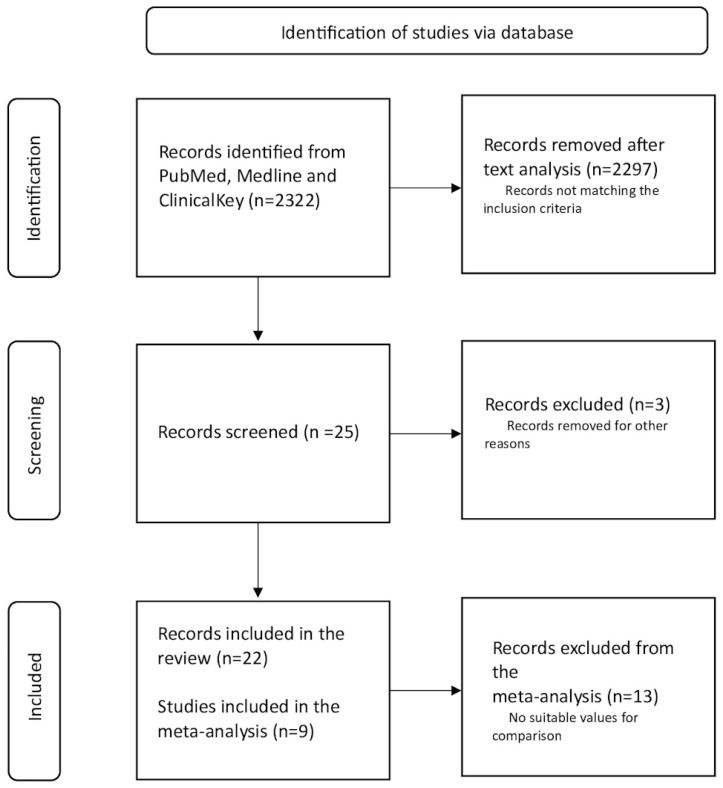
PRISMA flow diagram of the study selection process.

This study followed the guidelines of the Preferred Reporting Items for Systematic Reviews and Meta-analyses (PRISMA) protocol for conducting systematic reviews and meta-analyses normatively [55]. Figure 2 illustrates a PRISMA flow diagram of the study selection process for all articles.

The search for records concerning “ginseng and exercise”, “ginseng supplementation” and “ginseng supplements” resulted in 2322 articles, including three electronic databases. A total of 628 articles related to “ginseng and exercise”, 478 with ginseng supplementation”, and 1216 with “ginseng supplements” were obtained. Based on the full-text analysis, 2297 records were excluded. There were 12 articles repeated in Medline and PubMed in animal studies and 2 articles repeated in Medline in PubMed in human studies.

Search terms “ginseng and exercise”, “ginseng supplementation”, and “ginseng supplements” in the electronic database Medline yielded a total of 20 matching results, of which 13 were animal-related and 7 were human-related. The search terms “ginseng supplements” and “ginseng supplementation’ did not find any new matching records that did not appear after searching for “ginseng and exercise”.

A search of the electronic database PubMed yielded a total of 18 records matching this review. After their subsequent comparison with the Medline database, as many as 14 results that also appeared in this database were excluded. Ultimately, 4 new works about animals and 0 works about people were selected. As before, searching for “ginseng supplementation” and “ginseng supplements” in the PubMed database did not return any new results, which would not appear after viewing the results for “ginseng and exercise”. Some articles were excluded after the screening phase because the presentation of the data did not allow for a comparison with other papers. The final number of articles was 1.

The final search was the ClinicalKey electronic database. The total results that fit into the inclusion conditions amounted to 1. After checking the matching of the results with the two previous databases, 0 concerned with animal studies and 1 concerned with human studies were separated. As in the previous two cases, a search for “ginseng supplementation” and “ginseng supplement” did not release any new works that would not appear while searching for ginseng and exercise.

In total, 14 and 8 studies were selected, respectively, for animals and humans and included in the present review.

### 2.4. Risk of Bias

Cochrane’s risk-of-bias tool (RoB 2) was used to rate the methodological quality of the included studies [56]. Several areas were covered by this tool, e.g., insufficient outcome data, selective reporting of results, and other possible sources of bias. The application of RoB 2 for the included studies was performed by two separate authors, and all disagreements were resolved through discussion and consultation with a third party when necessary.

### 2.5. Statistical Analysis

Statistical analyses were conducted with the use of StatSoft software (version StatSoft version 1.0.67. www.statsoft.pl, accessed on 10 June 2022). The standardized effect size and 95% confidence intervals (95% CI) were calculated to weigh the results of the included studies. Considering acceptable heterogeneity status or homogeneity, the random-effect meta-regression model was used. The statistical significance was considered if *p* < 0.05.

## 3. Results

### 3.1. Analysis of Ginseng on the Selected Endurance Parameters in Animals

**Figure 3:**
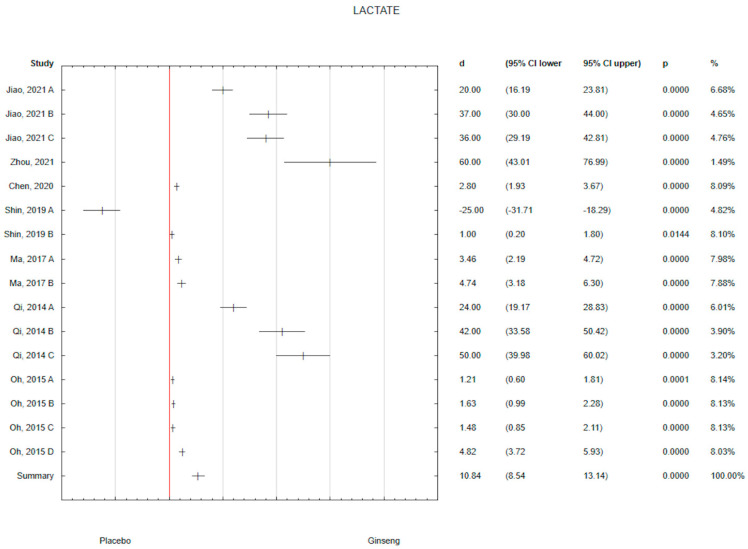
Forest plot of the results of the meta-analysis for ginseng effectiveness on lactate versus placebo group. Data are shown as mean effect sizes with 95% Cis [17,57,58,59,60,61,62].

Considering all studies, the effect of the overall interventions resulted in a significant influence of ginseng on the level of different biochemical parameters in animals. The analysis of the collected results showed a positive effect of ginseng on lactate level (d = 10.84; 95% CI 8.54 to 13.14) with *p* < 0.05 (Figure 3). The strongest effect of ginseng on lactate level was observed in Zhou’s study (d = 60.00; 95% CI 43.01 to 76.99), in which ginseng was administered for 14 days in a dose of 1.42 g/bw per day in the form of a water extract (Table 1). Taking into the relatively large percentage of data in the meta-analysis, the strongest effect statistically significant of ginseng on the level of lactate (d = 4.82; 95% CI 3.72 to 5.93) was observed in Oh 2015 D study, in which the dose was 10 mg/bw per day in the form of 20(S)-Protopanaxadiol and 20(S)-protopanaxatriol administered for 5 days (Figure 3). A similar strong significant effect was observed in the Mia 2017 B study (d = 4.74; 95% CI 3.18 to 6.30) in which for 4 weeks, a supplement in the form of Changbai Mountain Ginseng (CMG) Extract in the dose of 25 mg/kg per day was used (Figure 3).

**Figure 4:**
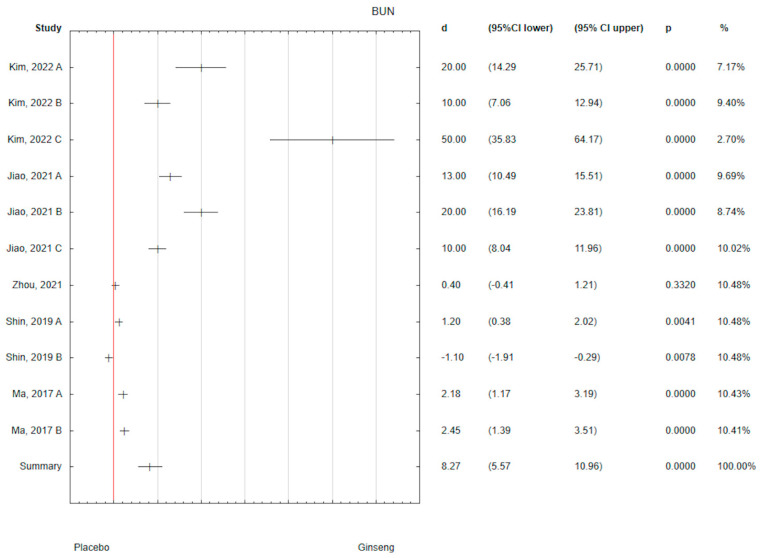
Forest plot of the results of the meta-analysis for ginseng effectiveness on BUN versus placebo group. Data are shown as mean effect sizes with 95% Cis [57,58,59,60,63].

The analysis of the collected results showed a positive effect of ginseng on the BUN level (d = 8.27; 95% CI 5.57 to 10.96) with *p* < 0.05 (Figure 4). The strongest effect of ginseng on BUN level was observed in Kim’s study (d = 50.00; 95% CI 35.83 to 64.17), which was administered for 30 days in a dose of 400 mg/bw in the form of extract (Table 1). Taking into the relatively largest percentage of data in the meta-analysis, the strongest effect statistically significant of ginseng on the level of BUN (d = 2.45; 95% CI 1.39 to 3.51) was observed in Ma 2017 B study, in which the dose was 25 mg/bw per day in the form of CMG Extract administered for 4 weeks (Figure 4).

**Figure 5:**
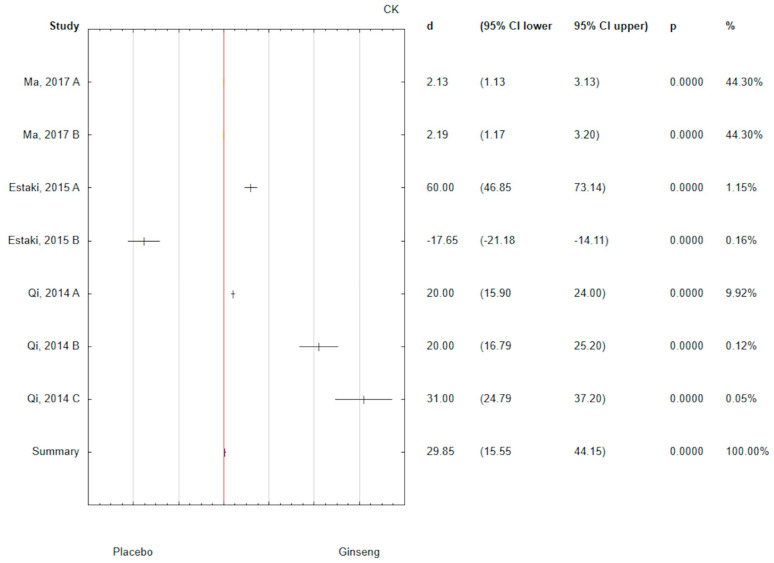
Forest plot of the results of the meta-analysis for ginseng effectiveness on CR versus placebo group. Data are shown as mean effect sizes with 95% Cis [60,61,64].

The analysis of the collected results showed a positive effect of ginseng on CK level (d = 29.85; 95% CI 15.55 to 44.15) with *p* < 0.05 (Figure 5). Taking into the relatively largest percentage of data in the meta-analysis, the strongest effect statistically significant of ginseng on the level of CK (d = 2.19; 95% CI 1.17 to 3.20) was observed in Ma 2017 B, in which the dose was 25 mg/bw per day administered for 4 weeks in the form of CMG Extract (Figure 5).

**Table 1:** All studies included in the meta-analysis (n = 9).

Author Year	Sample	Supplement Administration Protocol	Placebo Group	Duration	Form of Supplement	Parameter of Assessed Impact	Findings
Kim 2022 [63]	Mice (n = 50)	100 mg/kg per day, 200 mg/kg per day, 400 mg/kg per day or placebo	mice (n = 10)	30 days	Ginseng Extract	BUN	Administration of ginseng extract may have beneficial effects against physical fatigue during acute exercise.
Jiao, 2021 [57]	Mice (n = 30)	25 mg/kg per day, 50 mg/kg per day, 75 mg/kg per day or placebo	Mice (n = 10)	30 days	WSGP-S3 polysaccharide obtained from steamed ginseng via ultrafiltration	Lactate	WSGP-S3 might have a major contribution to the anti-fatigue effects of steamed ginseng polysaccharides.
						BUN	
Zhou, 2021 [58]	Rats (n = 8)	1.42 g/kg per day or placebo	Rats (n = 8)	14 days	Water extract ginseng (WEG)	Lactate	WEG exerts an anti-exercise-induced fatigue effect on a rat model of weight-loaded swimming.
						BUN	
Chen, 2020 [65]	Mice (n = 30)	25 mg/kg per day, 50 mg/kg per day, 100 mg/kg per day or placebo	Mice (n = 10)	3 weeks	20(S)-protopanaxadiol	Time of exercise	In the weight-loaded swimming test, daily PPD administration for 3 weeks increased mouse endurance swimming time.
Shin, 2019 [59]	Rats (n = 10)	0,1 mg/kg per day, 0,25 mg/kg per day or placebo	Rats (n = 5)	5 days	Panaxydol	Lactate	Panaxydol-treated groups showed significantly enhanced performance in forced swimming compared to control. In addition, a significant decrease in serum LDH level was found in panaxydol-treated group, while there were no alternations in levels of serum BUN and LAC.
						BUN	
Ma, 2017 [60]	Mice (n = 16)	5 mg/kg per day, 25 mg/kg per day or placebo	Mice (n = 8)	4 weeks	Changbai Mountain Ginseng (CMG) Extract	Lactate	CMG supplementation significantly improved swimming time to exhaustion in test animals; CMG has anti-fatigue activity; it decreased plasma lactate, ammonia, and CK and increased serum.
						BUN	
						CK	
Estaki, 2015 [64]	Rats (n = 20)	300 mg/kg per day of either an AL or AQ ginseng extract	Rats (n = 20)	14 days	Alcohol (AL) or aqueous (AQ) extract of North American ginseng	CK	Downhill running resulted in significant increases in plasma CK levels in placebo group; these changes were attenuated in both the AL and AQ-treated animals by 19% and 24%, respectively.
Qi, 2014 [61]	Mice (n = 36)	25 mg/kg per day, 50 mg/kg per day, 100 mg/kg per day or placebo	Mice (n = 12)	4 weeks	Ginsenoside Rb-1	Lactate	The blood lactate contents of mice in the test groups were significantly lower compared with the placebo group; the serum CK activities of mice in the 50 mg/kg per day and 100 mg/kg per day groups were significantly lower compared with the placebo group.
						CK	
Oh, 2015 [62]	Mice (n = 28)	5 mg/kg of PPD per day, 10 mg/kg of PPD per day, 5 mg/kg of PPT per day, 10 mg/kg of PPT per day or placebo	Mice (n = 8)	5 days	20(S)-Protopanaxadiol (PPD) and 20(S)-protopanaxatriol (PPT)	Lactate	Treatment of PPD and PPT attenuated the induction of serum levels of the biochemical parameters. In particular, treatment with PPT (10 mg/kg) led the serum levels to normal.

Research on ginseng shown in Table 2 also suggests that the clinical effect of the supplement can be obtained not only in relation to parameters, such as lactate, BUN, and CR, but also in several others, which are important in effective processes responsible for exercise exertion. Different research suggests the positive effect of ginseng as a longer time of exercise or decreased LDH level in comparison to placebo (Table 2). However, it can be noticed that the effect of the supplement may be dependent on the dose administered per body weight but also on the form of the supplement. In three studies (Zhou 2021; Estaki 2015 and Jiao 2022), a watered form of ginseng was administered, and usually, the dosages were much higher in comparison to the other forms of administration (Table 1 and Table 2).

### 3.2. Analysis of Ginseng on the Selected Endurance Parameters in Humans

Data collected from human studies conducted to date also indicate that ginseng may have a positive effect on the same parameters related to the physical effort that was analyzed in the studies conducted on animals (Table 3). In contrast, one study (Kate L. Pumpa, 2013) showed no convincing effect of ginseng supplementation on performance; the time of administration seems to be the shortest in comparison to the others. There is also one study (Hsin-Fu Lin, 2016) in which the time of administration is much shorter in comparison to the other studies lasting only one week time, but it was a complex of panax ginseng and salvia miltiorrhiza, which could influence the effect (Table 3).

## 4. Discussion

*Ginseng radix et rhizoma* is a pharmacopoeial material that has a long history in traditional medicine. It has been used for more than 2000 years, and it is considered a cure for all ailments. According to traditional Chinese medicine, ginseng strengthens one’s vitality, invigorates the spleen, replenishes one’s qi, and calms one’s nerves in the broadest context possible [79]. Much research confirms the multi-directional effect of ginseng, in particular, its regulatory effect on the nervous and endocrine systems and immune or digestive systems. In addition, previous and current in vitro and in vivo studies also prove that ginseng has beneficial effects on the cardiovascular system. Its mechanism of action includes antioxidant activity, control of vasomotor function, modulation of ion channels and signal transduction, improvement in lipid profiles, regulation of blood pressure, improvement in cardiac function, and reduction in platelet adhesion [80,81]. Numerous studies have found that ginseng plants were a rich source of biologically active compounds such as ginsenosides, gintonin, polysaccharides, peptides, polyacetylene, and glycoconjugates [82] that are responsible for pharmacological properties of ginseng, including anti-fatigue properties and an increase in physical performance. The phenomenon of fatigue and the mechanisms of its development/appearance are complex. The current theory of exercise-induced fatigue is that it is closely related to energy loss, specific metabolite accumulation, and oxidative stress [83].

In this research, an analysis of examining the anti-fatigue effect of ginseng on human and animal bodies was conducted based on the reports that were published between 2012 and 2023. The obtained results indicated that both humans and animals reacted by reducing fatigue after the use of ginseng supplements. The levels of tested parameters such as serum lactic acid, creatine phosphokinase (CK), or BUN were lower than those in the control, thereby proving that ginseng really could have an anti-fatigue effect. A similar observation was noticed in relation to VO2 max and time to exhaustion in human studies.

Serum lactic acid or creatine phosphokinase, besides blood urea nitrogen and lactic dehydrogenase, are important markers of body fatigue. Their levels were investigated after the administration of supplements other than those of the ginseng plant [84]. For example, ashwagandha and *Rhodiola rosea* significantly reduced the amount of CK in the blood following resistance exercise [85,86]. The Maca (*Lepidium meyenii Walp.*) extracts can remove accumulated metabolites, such as blood lactic acid and blood urea nitrogen, after weight-loaded forced swimming [87]. Analysis of the level of lactate in the blood, blood urea nitrogen, and the activity of creatine phosphokinase or lactic dehydrogenase as biochemical parameters after physical exercise allows for the assessment of metabolic stress, which accelerates the decline in muscle exercise capacity. These parameters influence the non-oxidative production and glycogenolysis of ATP, the metabolism of proteins and amino acids, the ability to produce ATP, and the removal of lactic acid in skeletal and heart muscles [84].

Many observations state that physical exercise may lead to a decrease in physical activity, disruption of energy homeostasis, and depletion of physical energy reserves, such as adenosine triphosphate (ATP), fat, or glycogen. Thus, the capacity to improve glycogen stores by increasing glycogen storage or delaying glycogen consumption in both the liver and muscles may effectively improve exercise endurance [88]. Such ability poses ginseng plants. Studies by Zhang et al. [89] and Zhou et al. [90] indicated that *P. ginseng* and *P. notoginseng* significantly increased the glycogen reserves of the liver and muscle; thereby, the supplementation of both ginseng species can enhance the anti-fatigue effect. The above data suggest that improved performance after ginseng supplementation may also be important for improving the energy metabolism of the heart muscle and indirectly contribute to protection against cardiovascular diseases, especially since *Panax* plants are also known to regulate or induce multiple cardiovascular protection mechanisms, including the regulation of various signaling pathways, which has been confirmed by both in vivo preclinical evaluation of Ginseng effect in animal models of cardiovascular diseases as well as some studies on in vitro cell lines and humans [91].

It was also proven that one of the protopanaxadiol derivatives—ginsenoside Rb1—improves fatigue syndrome by reducing skeletal muscle oxidative stress through activation of the PI3K/Akt/Nrf2 pathway in aged rats [92]. Likewise, the protective effect of Rb1 was observed in relation to heart muscles [93].

Furthermore, Rb1 and Rb2 upregulate myotube growth and myogenic differentiation by activating the Akt/mammalian target of rapamycin signaling and inducing myogenic conversion of fibroblasts, which may facilitate the muscle regeneration process [94].

Taking ginseng preparations may also indirectly increase the body’s efficiency through its antioxidant effect and the ability to scavenge free radicals. Free radicals include hydroxyl radical, superoxide anion radical, hydrogen peroxide, oxygen singlet, hypochlorite, nitric oxide radical, and peroxynitrite radical. They and other ROS are derived from metabolic processes. Under normal circumstances, generation and disposal are in dynamic balance. However, during an excessively vigorous exercise, the ROS that is quickly generated is unbalanced. Thus, the increase in free radicals can be the cause of oxidative stress, which can be regarded as a causal factor of muscle damage and body fatigue [84]. The literature data demonstrated that ginseng had strong antioxidant potential, and in vitro, antioxidant capacity was correlated with the plant’s age. It was also stated that diet supplementation of ginseng powders, particularly aged ginseng, markedly lowered lipid peroxidation and enhanced the antioxidant enzyme activities in various tissues such as heart, lung, kidney, and liver in animal models [72,73]. The antioxidant potential of ginseng has been proven based on the analysis of the free radical scavenging properties of individual active compounds of this plant. For example, saponin Rb1 increases GSH levels and decreases MDA content. In addition, Rb1 enhances antioxidant enzyme activity that reduces the MDA level in spinal cord-injured (SCI) rats by stimulating the Nrf2/HO-1 signaling pathway. Ginseng metabolites indicate that ginseng administered before physical exercise can alleviate the damage caused during training. Ginseng has a systemic effect, which indicates its adaptogenic properties and the ability to regulate the action of all organs under the influence of stress conditions.

It is worth emphasizing that the effects of ginseng may depend on the dose of the preparation, the method of administration, or its pharmaceutical form. In the analyzed studies, the pharmaceutical forms of supplements were different. They included both water and alcohol extracts and powdered roots or single active ingredients. The supplement administration and posology were also different. European Union herbal monograph on *Panax ginseng* C.A. Mey, radix states that a single dose of powdered herbal substance should be 250–1200 mg (daily dose 600–2000 mg) for adults and the elderly. When using extracts, a single dose depends on the method of preparation of the extracts and can range from 40 mg to 360 mg (daily: 40–670 mg) for dry extracts. Different single and daily doses are listed for soft extracts (single dose: 219–440 mg, daily dose: 440–700 mg) and still others for liquid extracts (single dose: 500 mg–9.9 g; daily dose: 900 mg–19.8 g) [95].

## 5. Strengths and Weaknesses

The present review has several strengths. Firstly, according to our knowledge, it is the first systematic review with meta-analysis that addressed the effects of ginseng supplementation on physical performance in the context of supporting CVD patient management. Secondly, the present investigation includes a comprehensive exposure of the available literature and a careful appraisal of its value, and it was prepared to be in line with the current PRISMA recommendations. Also, the conducted analysis showed that ginseng could potentially have a positive effect on parameters related to the body’s efficiency in both animal and human models. On the other hand, it was revealed that the procedures for administering supplements and the frequency and duration of their use are different, which may affect the obtained results. The above indicates that more in-depth research on ginseng supplementation is still needed.

However, some limitations have to be emphasized. The number of studies included in the meta-analysis was relatively small. Unfortunately, some of the results from the selected research were presented only in the figures without the average values that were required for the meta-analysis. Also, the results were often given in units or parameters that could not be compared. In addition, the research process was limited to articles in English, which could introduce bias.

## 6. Conclusions

We attempted to review and analyze the results of ginseng supplementation in terms of its effect on exercise endurance. A review of the literature and conducted a meta-analysis identified that ginseng supplementation could have a positive effect on exercise endurance, regulating the level of lactate, BUN, or CK in the blood. Also, a few studies suggest the beneficial role of ginseng supplementation in the improvement in VO2 max. Due to the fact that most of the studies were based on animal models, further research on human models is needed to identify the most effective dosage or form of applied ginseng to be a supportive element in CVD management.

## Figures and Tables

**Table 2:** Supplement effectiveness review based on the included articles on animals (n = 5).

Author Year	Sample	Supplement Administration Protocol	Duration	Form of Supplement	Parameter of Assessed Impact	Significant Results
Ju Jin, 2023 [66]	Mice	200 mg/kg per day, 400 mg/kg per day or placebo	9 weeks	The ginseng (*Panax ginseng* C. A. Meyer, Korea) berry concentrate (GBC)	Lactate, time of exercise, LDH,	The results from our current study suggest that GBC may benefit metabolism, mitochondrial function, and muscle physiology.
Shin, 2020 [67]	Mice (n = 8)	100 mg/kg per day or placebo	28 days	Red ginseng extract (RG)	Time of exercise	Specific ginsenosides of red ginseng can contribute to the improvement in exercise endurance and muscle function.
Lee, 2013 [68]	Rats (n = 16)	25 mg/kg per day, 50 mg/kg per day, 100 mg/kg per day or placebo	8 weeks	IH901 (a ginsenoside intestinal metabolite)	Time of exercise, LDH, CK	IH901 consumption in aging rats after eccentric exercise has beneficial effects on anti-inflammatory and anti-oxidant activities.
Jiao, 2022 [69]	Mice	200 mg/kg or placebo	8 weeks	Water-soluble ginseng polysaccharide’s acidic fraction (WGPA)	Lactate, LDH, CK	Findings depict a previously unappreciated role in the recovery of exercise-induced fatigue by ginseng pectin.
Lim, 2022 [70]	Mice (n = 16)	6 mg/kg per day, 12 mg/kg per day or placebo	4 weeks	Ginsenoside Rf (G-Rf)	Time of exercise	G-Rf treatment increases the production of energy required for exercise and consequently increases exercise endurance.

**Table 3:** Supplement effectiveness review based on the included articles on humans (n = 8).

Author Year	Sample	Supplement Administration Protocol	Duration	Form of Supplement	Parameter of Assessed Impact	Significant Results
Ching-HungLin, 2021 [71]	Men (n = 12)	AG capsules (4 × 400 mg AG extract) or placebo capsules (4 × 400 mg hydroxymethylcellulose) once a day	2 × 30 days with the washout period lasting more than 7 days	AG extract or hydroxymethylcellulose in the same capsule volume (4 × 400 mg). (major bioactive ginsenosides present in the AG extract were Rb1 (8.67%, *w*/*w*), Rc (0.99%, *w*/*w*), Rd (1.05%, *w*/*w*), and Re (5.08%, *w*/*w*)	CK	The average CK activity at POST-24 H increased to 223% and 191% by supplementation P (*p* = 0.002 and *r* = 0.62) and AG (*p* = 0.003 and *r* = 0.61), respectively, compared with the PRE-EX level. The CK activity returned to the pre-DH running level after AG supplementation and not after P supplementation at 48 and 72 h after DH running (*p* = 0.003 and *r* = 0.61 for POST-48 h *p* = 0.006 and *r* = 0.56 for POST-72 H).
Yi-Ming Chen, 2021 [72]	Females (n = 20)	Four capsules × 500 mg each of the GS extract and placebo (2 g total per day) two times a day (08:00 and 18:00 h) with glucose water.	6 weeks	Capsule (GS root powders)	CK (U/L)	CK showed a significant decrease of 34.20% (*p* = 0.0001) compared with the placebo group.
					TTE	GS group showed a significant 1.53-fold increase in the exhaustion time compared with the placebo group (15.1 ± 3.0 min and 9.9 ± 1.7 min, respectively, *p* = 0.0001).
					VO2 max	GS—a 1.38-fold higher oxygen consumption compared to placebo (2.5 ± 0.4 L min^−1^ and 1.8 ± 0.3 L min^−1^, respectively, *p* = 0.0005). Alternative formulation showed a 1.43-fold higher value in the GS group compared with the placebo group (44.3 ± 4.2 mL kg^−1^ min^−1^ and 30.9 ± 5.1 mL kg^−1^ min^−1^, respectively, *p* < 0.0001).
Kate L. Pumpa, 2013 [73]	Men (n = 20)	Four capsules (4 × 1000 mg) 1 h prior to the downhill run, then another 4 immediately after the downhill run. Subjects then consumed 4 capsules every 4 waking hours for the first 48 h and then consumed 4 capsules twice daily for the remaining 48 h of this study.	5 days	Capsule with powder		Considering all data from this study, P. notoginseng did not convincingly have an effect on performance, muscular pain, or assessed blood markers in well-trained males after an intense bout of eccentric exercise that induced DOMS.
Chien-Wen Hou, 2015 [74]	Men (n = 26)	One capsule night before exercise and 1 capsule 1 h prior to the exercise (2 × 5 mg of Rg1 with flour powder or placebo only flour powder).	3 trials with 4-week washout period	Capsule with powder	time to exhaustion (min)	Rg1 significantly increased exercise time to exhaustion (Rg1: 38.3 ± 6.7 min versus Placebo: 31.8 ± 5.0 min) during a cycle ergometer test at 80% VO2 max_._
					Total work	Ginseng: 306 ± 55 *p* = 0.04 Placebo: 254 ± 41 *p* = 0.04
Jinfu Wu, 2018 [75]	Men (n = 12)	Rg1 (5 mg) 1 h before the test	Acute intake (crossover study with a 4-week washout period between each trial)	Ginseng component Rg1 (5 mg)	TTE	Rg1 supplementation significantly increased cycling time to exhaustion by 12% (PLA: 1219 ± 135 s vs. Rg1: 1364 ± 145 s, *p* < 0.05)
					Power output	Increased power output by 13% (PLA: 199 ± 31 kJ vs. Rg1: 225 ± 33 kJ, *p* < 0.05). Eight of 12 participants with the Rg1 (5 mg) trial showed significantly improved cycling time compared with the PLA trial.
Bei Yan, 2018 [76]	Men (n = 21)	10 capsules per day	30 days	Capsule (500 mg of whole-root Korean ginseng powder) Rg1 (54 747 µg/g), Rc (37 500 µg/g), Rb1 (8597 µg/g), Re (3348 µg/g), Rf (1821 µg/g), Rb2 (1455 µg/g), Rd (636 µg/g), Rg3 (156 µg/g), Rh1 (163 µg/g), and Rg2 (77 µg/g)	CK	After 15 days, in the control group, there was a +3.48-fold change, and in the GS group, there was a +2.47-fold change.
					BUN	After 15 days in the control group, there was a +1.32-fold change, and after 30 days, +1.24-fold change. In the test group, after 15 days, there was a +1.24-fold change.
Eon Sook Lee, 2017 [77]	N = 117, (completed n = 81)	UG0712 250 mg twice/d in high-dose ginsenoside group, UG0712 50 mg twice per day in low-dose ginsenoside group, or CMC 250 mg twice/d in placebo group	12 weeks	Capsules with powder; UG0712 contained partially hydrolyzed ginseng leaf extract.	VO2 max	ITT set: significantly improved (from 28.6 ± 4.9 to 33.7 ± 4.9 mL/kg/min, *p* < 0.001 in high-dose group (n = 39), from 29.1 ± 4.7 to 33.3 ± 6.0 mL/kg/min, *p* = 0.005 in low-dose group (n = 39). The RM analysis of covariance with Dunnett’s multiple comparison tests showed the difference in VO2 max increase in baseline of three groups at each step. VO2 max increase in baseline of the high-dose group was significantly higher than the placebo group after adjusting for baseline VO2 max (*p* = 0.029; PP set: High dose from 28.6 ± 5.1 to 34.5 ± 4.4; Low dose from 28.0 ± 3.5 to 33.7 ± 5.8.
Yi Yang, 2021 [78]	Healthy people (n = 110) (completed n = 107)	2× day, 3 tablets each time with warm water	8 weeks	Hard capsule, contents are yellowish–brown, product specification: 0.465 g/granule × 90 granule/bottle, total saponin content 3.5–4.8 g/100 g.	Blood lactate	In self-comparison, the blood lactate content in the test group after the test was significantly lower than that before the test (*p* < 0.01); Compared between the groups after the test, the blood lactate content in the test group was significantly lower than that in the control group (*p* < 0.05). Test group before the test: 214.52 ± 30.22, and after the test: 199.17 ± 34.80
					CK	The content of CK in the test group after the test was significantly lower than that before the test (*p* < 0.05) in the same group. The content of CK in the test group was significantly lower than that in the control group (*p* < 0.05). Test group before the test: 175.20 ± 38.42, and after the test: 163.30 ± 26.84
